# Flow cytometry dataset for cells collected from touched surfaces

**DOI:** 10.12688/f1000research.8338.2

**Published:** 2016-10-07

**Authors:** Ye Jin Kwon, Cristina E. Stanciu, M. Katherine Philpott, Christopher J. Ehrhardt

**Affiliations:** 1 Department of Forensic Science, Virginia Commonwealth University, Richmond, USA

**Keywords:** forensic science, flow cytometry, epithelial cell, touch mixtures

## Abstract

‘Touch’ or trace cell mixtures submitted as evidence are a significant problem for forensic laboratories as they can render resulting genetic profiles difficult or even impossible to interpret. Optical signatures that distinguish epidermal cell populations from different contributors could facilitate the physical separation of mixture components prior to genetic analysis, and potentially the downstream production of single source profiles and/or simplified mixtures.  This dataset comprises the results from antibody hybridization surveys using Human Leukocyte Antigen (HLA) and Cytokeratin (CK) probes, as well as surveys of optical properties of deposited cells, including forward scatter (FSC), side scatter (SSC), and fluorescence emissions in the Allophycocyanin (APC) channel.  All analyses were performed on “touch” samples deposited by several different contributors on multiple days to assess inter- and intra-contributor variability.

## Introduction

Flow cytometry has proven a viable approach for differentiating cell populations in many types of uncompromised (i.e. non-degraded) forensic mixture sample ( Dean *et al.*, 2015; Schoell *et al.*, 1999; Verdon *et al.*, 2015). However, application to ‘touch’ or trace epithelial cell mixtures remains a challenge since many cell surface features are lost or obscured during the process of keratinocyte differentiation, leaving few biochemical or structural features in shed corneocytes that vary between individual contributors. Preliminary research has identified specific optical characteristics—namely, red autofluorescence and forward scatter (FSC) and side scatter (SSC) profiles—that may vary between touch samples deposited by different contributors and collected immediately after deposition ( Stanciu *et al.*, 2016). For this dataset, we built on these studies by examining optical properties such as these on an additional two flow cytometry platforms (including different settings, such as channel voltages). We also investigated the capacity of touch epithelial cells to bind to two different classes of antibody probes: Human Leukocyte Antigen (HLA), which has been successfully utilized to separate uncompromised mixtures of blood and other bodily fluids (e.g. Dean *et al.*, 2015) and Cytokeratin (CK), which is known to be a dominant component of epidermal cells. This dataset includes samples that were collected and analyzed immediately after deposition (i.e. ‘fresh samples’), as well as samples that were collected up to seven days after deposition.

## Methods

Touch epithelial samples Flow cytometry source data for all samples are provided in Flow Cytometry Standard (.fcs) format files. Source data files are organized into three different repositories labeled, “HLA Hybridization”, “Cytokeratin Hybridization”, and “Autofluorescence”. Flow cytometry data collected on aged touch samples is contained within the Autofluorescence data folder. File names are labeled with the anonymized sample ID number, date of analysis, and the instrument platform used. Source data files are also labeled by the specific probe used where applicable (e.g., AE1, HLA-A02, etc...) as well as by the voltage setting when different from those described in the above Methods. Click here for additional data file. Copyright: © 2016 Kwon YJ et al. 2016 Data associated with the article are available under the terms of the Creative Commons Zero "No rights reserved" data waiver (CC0 1.0 Public domain dedication).

Touch samples were collected from six volunteers using the following protocol which was approved by the VCU-IRB (#HM20000454_CR). Contributors either rubbed a sterile polypropylene conical tube (P/N 229421; Celltreat Scientific) for five minutes using their entire hand (i.e. palm and fingers), or held a tube in their hands (no rubbing) for five minutes. Cells were then immediately collected from the surface with six sterile pre-wetted swabs (P/N 22037924; Fisher Scientific) followed by two dry swabs or were collected between 12 hours and seven days after deposition.

Cells were also deposited onto a wooden kitchen knife handle and the grip of a resin mold of a handgun by handling each substrate for five minutes. Several contributors donated samples on multiple days to assess intra-contributor variation of epithelial cell populations.

To elute the collected cells into solution, the swabs were manually stirred then vortexed for 15 seconds in 10 mL of ultrapure water (18.2 MΩ∙cm).

### Antibody hybridization

For antibody hybridization experiments, three milliliters of cell solution were centrifuged at 5,000×g for five minutes. The supernatant was decanted and the pellet was resuspended in 100 µl PBS buffer and 1 µL of Human Fc Receptor block (Cat# 130-059-901, Miltenyi Biotec) to increase the specificity of antibody binding before reaction with either HLA or CK probes. This suspension was allowed to incubate at room temperature for 10 minutes.

For HLA hybridizations, cells were then incubated with one of three different antibody probes for 30 minutes: mouse anti-human monoclonal antibody (mAb) HLA-ABC-FITC (Cat# 311403, BioLegend), HLA-A02-FITC, (Cat#343303, Biolegend), HLA-B7-FITC, (Cat#sc-53304, Santa Cruz Biotechnology). Cells incubated with anti-mouse IgG2a-FITC (Cat# 343303, BioLegend) for 30 minutes served as the isotype control for this set of hybridizations. Cells were then pelleted and washed once in 1x FACS buffer [PBS supplemented with 2% Fetal Bovine Serum (FBS, Cat# 100-106, Gemini BioProducts) and 10% Sodium Azide (Cat# S2002, Sigma-Aldrich)] and re-suspended in the same FACS buffer solution until flow cytometry analysis.

For CK hybridization experiments, cells were incubated with either cytokeratin probe ‘AE1’ (recognizes CKs 10, 14, 15, 16 and 19; Cat# 14-9001-80, Affymetrix eBioscience) or ‘AE3’ (recognizes CKs 1, 2, 3, 4, 5, 6, 7, 8), Cat# 14-900-80, Affymetrix eBioscience) for 30 minutes followed by reaction with a secondary antibody, anti-mouse IgG1-APC (Cat# 17-4015-80, Affymetrix eBioscience). We used anti-mouse IgG1-APC (Cat#17-4714-42, Affymetrix eBioscience) to create the isotype control for these experiments, incubating for 30 minutes. As before, cells were washed once and then resuspended in 1xFACS buffer prior to analysis.

### Flow cytometry

Cell solutions—both those treated with antibody probe and those that were not—were passed through 100 µm filter mesh prior to flow cytometry. Flow cytometric analysis of cells was performed on three different platforms: BD FACSCanto™ II Analyzer, BD FACSAria™ II High-Speed Cell Sorter, and BD Influx™ Cell Sorter (all from Becton Dickinson and Company, Franklin Lakes, NJ, USA). The FACSCanto and FACSAria platforms were equipped with 488nm and 633nm lasers. The Influx cell sorter was equipped with 488 561, and 640nm lasers.

For HLA and CK studies, flow cytometry analysis was performed on the BD FACSCanto™ II Analyzer. Channel voltages were set as follows: Forward Scatter (FSC, 150V), Side Scatter (SSC, 200V), Alexa Fluor 488 (FITC, 335V), Phycoerythrin (PE, 233V; PE-Cy5, 300V; PE-Cy7, 400V), and Allophycocyanin (APC, 250V).

Autofluorescence studies of touch samples were performed on one of two BD FACSAria™ II flow cytometers, or on the BD Influx Cell Sorter (additionally, analysis of unstained samples conducted on the FACSCanto, at the settings described above, are the equivalent of autofluorescence studies). Channel voltages for the FACSAria were set as follows: FSC, 200V; SSC, 475V; and APC, 400V. Channel voltages for the BD Influx were set to the following: FSC, 17.5V; SSC, 16V; and APC, 74.6V.

## Dataset content

Flow cytometry source data for all samples are provided in Flow Cytometry Standard (.fcs) format files. Source data files are organized into three different repositories labeled, “HLA Hybridization”, “Cytokeratin Hybridization”, and “Autofluorescence”. Flow cytometry data collected on aged touch samples is contained within the Autofluorescence data folder. File names are labeled with the anonymized sample ID number, date of analysis, and the instrument platform used. Source data files are also labeled by the specific probe used where applicable (e.g., AE1, HLA-A02, etc...) as well as by the voltage setting when different from those described in the above Methods.

## Data availability

The data referenced by this article are under copyright with the following copyright statement: Copyright: © 2016 Kwon YJ et al.

Data associated with the article are available under the terms of the Creative Commons Zero "No rights reserved" data waiver (CC0 1.0 Public domain dedication). 

F1000Research: Dataset 1. Touch epithelial samples, 10.5256/f1000research.8338.d137992 ( Kwon *et al.*, 2016).
