# Supplementary material for: Flow cytometry dataset for cells collected from touched surfaces
Source: F1000Res. 2016 Oct 7;5:390. Originally published 2016 Mar 23. [Version 2] doi: 10.12688/f1000research.8338.2 (PMC5200940; doi:10.12688/f1000research.8338.2)
Supplement: Peer review history

# Peer review: Referee response for version 2

**DOI:** 10.5256/f1000research.10476.r16849

**Published:** 2016-12-28

**Authors:** Dieter Deforce

**Affiliations:** 1 Laboratory for Pharmaceutical Biotechnology, Ghent University, Ghent, Belgium

The authors have provided additional data making the dataset more relevant for a forensic setting.

I have read this submission. I believe that I have an appropriate level of expertise to confirm that it is of an acceptable scientific standard.

# Peer review: Referee response for version 2

**DOI:** 10.5256/f1000research.10476.r16850

**Published:** 2016-10-07

**Authors:** Peter K Rogan

**Affiliations:** 1 Dept of Biochemistry, Schulich School of Medicine and Dentistry, University of Western Ontario, London, ON, N6A 5C1, Canada

The authors have addressed my comments with additional data. The analysis of this data is ongoing, and not included in this paper.

I have read this submission. I believe that I have an appropriate level of expertise to confirm that it is of an acceptable scientific standard.

# Peer review: Referee response for version 1

**DOI:** 10.5256/f1000research.8964.r13496

**Published:** 2016-04-26

**Authors:** Peter K Rogan

**Affiliations:** 1 Dept of Biochemistry, Schulich School of Medicine and Dentistry, University of Western Ontario, London, ON, N6A 5C1, Canada

The authors describe a dataset used for flow cytometric analysis of sloughed epithelial cells from a set of 6 individuals. It is not at all clear why these data are different from those reported in their copublished research note ( http://f1000research.com/articles/5-180/v1) which provides an analysis of these data. The anonymized names of the individuals in the two papers are the same and they are both in .fcs format. The Data Note is larger than the data in the Research Note, presumably because it contains technical replicates. The differences are trivial. In fact, the Research Note would have greater validity if the Data Note dataset was incorporated into this document and discussed there.

The second point that should be addressed is how the statistics of the fluorescence distributions (FS, SS) for different samples from the same individual or from different individuals can be compared given that the intensities vary, presumably as a result of the differences in yield from each sample. The reliability of using these histograms for making comparisons between replicates or individuals for forensic or any other applications could be suspect (eg. sample R12) due to noise, broad distributions or other factors. I suggest that the authors determine and provide minimum threshold criteria for analysis of a sample or comparison with other samples.

I have read this submission. I believe that I have an appropriate level of expertise to state that I do not consider it to be of an acceptable scientific standard, for reasons outlined above.

# Response

**Published:** 2016-09-29

**Authors:** Christopher Ehrhardt

**Affiliations:** Virginia Commonwealth University, USA

We agree that the differences between this manuscript and another publication currently in review at F1000 research could have been clearer. The cited manuscript ( http://f1000research.com/articles/5-180/v1) was a preliminary survey of autofluorescence using one flow cytometry platform, the BD FACSCanto II Analyzer, whereas this study used two additional flow cytometery platforms, the BD FACSAria II cell sorter and the BD Influx Cell Sorter; different channel voltages were applied on each of the three flow cytometers, and the Influx is equipped with a different set of excitation lasers than the Aria and Canto. Additionally, for each manuscript a separate, non-overlapping set of touch samples was analyzed, although the reviewer is correct that some of the same donors contributed samples to both studies, albeit collected on different days. Given observed differences in fluorescence profiles for some contributors from one day to the next, sampling from the same contributor over multiple days adds to our understanding of this phenomenon.

 To further distinguish this Data Note from previous publication we have significantly revised the Data Note to include a more comprehensive set of samples and experimental conditions, investigating both optical properties and antibody binding capacities of ‘touch’ epithelial cell populations. This includes flow cytometry data for autofluorescence as well as antibody hybridizations with HLA and Cytokeratin probes. As the reviewer suggests, we are also in the process of incorporating aspects of this dataset into other manuscripts that discuss the biochemical and forensic applications of flow cytometry analysis of touch samples (e.g., pre-print available at http://biorxiv.org/content/early/2016/03/28/045948).

 We also agree with the reviewer's second point that there may be significant variability in the level of autofluorescence exhibited by touch samples. This in turn may be influenced by a variety of factors such as presence or absence of exogenous compounds, and/or intrinsic biological characteristics of the cells themselves. Understanding this variability, particularly for samples derived from the same contributor or for aged/degraded samples, is necessary to assess the potential utility of flow cytometry-based cell separation techniques such as FACS for downstream DNA profiling of separated cell populations derived from ‘touch’ biological mixtures.

 We would note that although differences in cell yield change the *number *of cells (Y axis) fluorescing in the red portion of the spectrum at a given RFU value (X axis), it is nonetheless possible to develop a sense of how the average *intensity* of red autofluorescence exhibited by cells collected from different individuals, or from the same individual on different days, varies by comparing histograms, regardless of cell yield.  We agree that an individual’s total cellular contribution to a biological mixture will ultimately be an important factor in whether that individual’s DNA profile can be successfully generated from a sorted mixture.  However, any kind of minimum cell count threshold would be inextricably linked to the sensitivity/efficiency of downstream DNA typing methodologies used on sorted cell populations, and are beyond the scope of this Data Note.

# Peer review: Referee response for version 1

**DOI:** 10.5256/f1000research.8964.r13031

**Published:** 2016-04-19

**Authors:** Dieter Deforce

**Affiliations:** 1 Laboratory for Pharmaceutical Biotechnology, Ghent University, Ghent, Belgium

The article suggests "touch samples" however the data set contains no data on forensic relevant touch samples. The six samples were from volunteers rubbing their entire hand. These are "fresh" cells and might not show the same flow characteristics as cells left behind on an object after a touch contact and having "aged" on the object. The dataset provided is as it is not really relevant to the forensic field. 

In addition I think that 6 samples is maybe a too limited number for this kind of studies.

I have read this submission. I believe that I have an appropriate level of expertise to confirm that it is of an acceptable scientific standard, however I have significant reservations, as outlined above.

# Response

**Published:** 2016-09-29

**Authors:** Christopher Ehrhardt

**Affiliations:** Virginia Commonwealth University, USA

We agree with the reviewer that there is an important distinction between ‘fresh’ biological samples and ones that are aged and/or degraded since the latter is more likely to be encountered in forensic caseworking samples. Uncompromised samples can nonetheless provide an important foundation for future studies that explicitly examine the effects of aging/degradation on optical signatures identified by the initial study. We also note that there are forensic scenarios where fresh touch samples may be collected and analyzed for an investigation. For example, a firearm may be discarded by a suspect and immediately collected during pursuit by law enforcement.

 However, to increase the forensic relevance of this dataset the new version of the manuscript includes samples that were aged between 12 hours and seven days before collection, as well as samples that were collected from forensically relevant substrates such as replica firearms and knife handles.

 For the updated version of the manuscript we have also expanded the dataset to include 33 different contributors.
